# Silver-coated magnetic nanocomposites induce growth inhibition and protein changes in foodborne bacteria

**DOI:** 10.1038/s41598-019-53080-x

**Published:** 2019-11-25

**Authors:** Seong B. Park, Shecoya B. White, Christy S. Steadman, Tibor Pechan, Olga Pechanova, Henry J. Clemente, Rooban V. K. G. Thirumalai, Scott T. Willard, Peter L. Ryan, Jean M. Feugang

**Affiliations:** 1 Department of Animal and Dairy Sciences, Mississippi State University, Mississippi State, MS 39762 USA; 2 Department of Food Science, Nutrition and Health Promotion, Mississippi State University, Mississippi State, MS 39762 USA; 3 Institute for Genomics, Biocomputing and Biotechnology, Mississippi State University, Mississippi State, MS 39762 USA; 4 Clemente Associates, Inc., Madison, CT 06443 USA; 5 Institute for Imaging and Analytical Technologies, Mississippi State University, Mississippi State, MS 39762 USA; 6 Department of Biochemistry, Molecular Biology, Entomology and Plant Pathology, Mississippi State University, Mississippi State, MS 39762 USA; 7 Department of Pathobiology and Population Medicine, Mississippi State University, Mississippi State, MS 39762 USA

**Keywords:** Nanobiotechnology, Antimicrobials

## Abstract

Cytotoxicity concerns of nanoparticles on animal or human bodies have led to the design of iron oxide core nanocomposites, coated with elemental silver to allow their magnetic removal from bio-mixtures. Although the antimicrobial effect of silver is well-described, the effects of nanoparticles derived from silver on microorganisms remain unfolded. Here, we characterized a customized magnetic silver nanocomposite (Ag-MNP) and evaluated its effects on bacterial growth and protein changes. The Ag-MNP displayed both longitudinal and round shapes under High-Resolution Transmission Electron Microscopy imaging, while the Energy Dispersive X-ray Spectroscopy and X-ray diffraction analysis confirmed the presence of Ag, Fe_3_O_4_ (Magnetite) and FeO_2_ (Goethite). Optical density, bioluminescence imaging, and Colony Forming Unit assessments revealed that the presence of Ag-MNP induced strong dose-dependent bacteria (*Escherichia coli* O157:H7, *Salmonella enterica* serovar Typhimurium and *S*. Anatum) growth inhibition. The TEM imaging showed penetration and infiltration of bacteria by Ag-MNP, leading to membrane degeneration and vacuole formation. The presence of Ag-MNP led to fifteen up-regulated and nine down-regulated proteins (P < 0.05) that are involved in cell membrane synthesis, inhibition of protein synthesis, interference with DNA synthesis, and energy metabolism inhibition. This study provides insights to develop alternative antimicrobials to treat foodborne pathogens with antibiotic resistance avoidance.

## Introduction

Multi-drug resistance (MDR) has been a major concern in combating infectious diseases. The MDR has disseminated not only in pathogenic bacteria but also in environmental microorganisms due to the abuse of antibiotics and widespread MDR genes^1^. Consequently, MDR bacteria have become less- or non-susceptible to various antibiotics by the alteration of either target protein, membrane permeability, escalation of efflux proteins and degradation of antibiotics^2^. The development of alternative antimicrobial agents has become quite urgent since some pathogenic bacteria including methicillin-resistant *Staphylococcus aureus* and vancomycin-resistant *Enterococci* show no susceptibility to existing antibiotic therapy^3,4^. The improvement of optic, electronic, catalytic, and physio-chemistry properties of many nanomaterials (gold^5,6^, silver^7^, carbon^8^, triclosan-bound silica^9^, zinc^10^, titanium^11^, copper^12^, and ferric oxide^13^) have shown significant antimicrobial potentials^14,15^.

Ferric oxide nanoparticles display antimicrobial activity by impacting reactive oxygen species (ROS), causing physical and mechanical damages on bacteria^13^. The amino-substituted pyrimidines presented on gold nanoparticles showed antibiotic action against MDR bacteria via disruption of the bacterial cell membrane^6^. Additionally, gold nanoparticles capped with vancomycin showed enhanced bactericidal effects against vancomycin-resistant *Escherichia coli* by inhibition of polyvalent activity on the bacterial surface^5^. Among these nanomaterials, silver nanoparticles have shown a broad spectrum of sterilization and antimicrobial properties against more than 650 bacteria strains^16^, justifying the range applications for wound dressing, medical catheter, water purification, cosmetics, clothing, medical ointment, and packaging^17,18^. Studies have shown that silver nanoparticles interact directly with bacteria membrane through protein thiol (-SH) groups and penetrate the bacteria to cause loss or membrane integrity and organelle damages^19^; however, their ability to generate ROS causing oxidative stress and toxicity of both prokaryote and eukaryote cells^20,21^, remains a concern for further bio-applications. Therefore, the need to develop alternatives to decrease or eliminate such cytotoxicity becomes crucial.

Iron oxide nanoparticles have the potential for selective separation of particles by external magnetic fields with highly attractive properties. These nanoparticles have been used for pollutant removal in wastewater^22^ and various bio-applications (i.e., magnetic resonance imaging, targeted drug delivery, magnetic separation of immune cells and tissue engineering) have been reported^23–26^. Silver-conjugated magnetic iron oxide nanocomposites (Ag-MNP) have shown antibacterial properties while being environmentally friendly, due to their durability and recyclability^27^; however, the underlying mechanism of actions of Ag-MNP remain unfolded.

In the present study, synthesized Ag-MNP nanocomposites were characterized by Transmission Electron Microscopy (TEM), Energy Dispersive X-ray Spectroscopy (EDS) and X-ray diffraction (XRD) followed with the evaluation of their antimicrobial activities through growth kinetic analysis using optical density and *in vivo* bioluminescent imaging techniques. The interaction between Ag-MNP and bacteria was observed by TEM and its cytotoxicity was assessed through cell membrane integrity measurement of HEP-2 cells. Finally, the molecular mechanism of Ag-MNP was assessed through a gel-based proteomic approach.

## Results

### Morphological and compositional analysis of silver-coated magnetic nanocomposites

**Figure 1:**
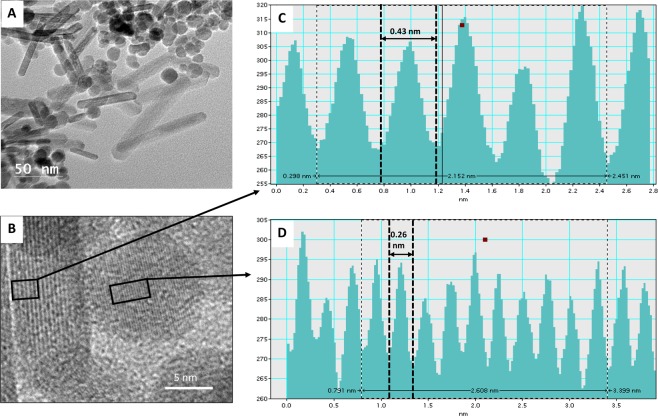
High-resolution transmission electron microscopy (HRTEM) analysis of silver-coated magnetic nanocomposites (Ag-MNP). (**A**) Rod and spherical shapes of Ag-MNP. (**B**) HRTEM micrograph of Ag-MNP. Line intensity profiles of rod type (**C**) and spherical type (**D**).

**Figure 2:**
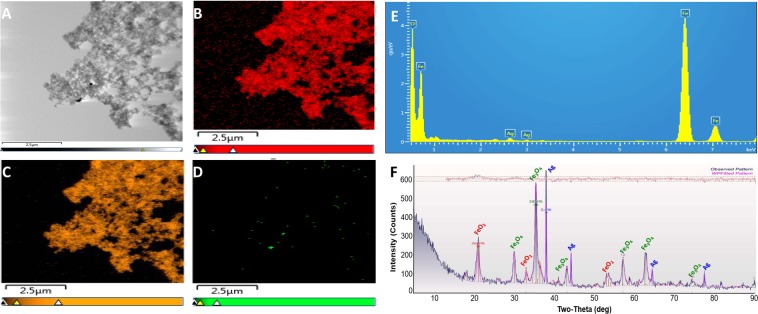
Energy dispersive spectroscopy (EDS) analysis and X-ray diffraction (XRD) of silver-coated magnetic nanocomposites (Ag-MNP). The TEM image of Ag-MNP is shown in (**A**). The EDS mapping of Ag-MNP shows iron, oxygen, and silver distributions in micrographs (**B**–**D**), respectively. The EDS spectrum reveals the main components (iron, oxygen and silver) of the Ag-MNP (**E**). The X-Ray Diffraction (XRD) pattern analysis of Ag-MNP indicates the presence of silver, Magnetite (Fe_3_O_4_) and Goethite (FeO_2_) in Ag-MNP (**F**).

The HR-TEM imaging of synthesized Ag-MNP nanocomposites revealed both rod and spherical shapes (Fig. 1A). The rod shape showed width and length averages of 19 nm and 23 to 127 nm, respectively, while the spherical shape had an average diameter of approximately 17 nm. Rod and spherical Ag-MNP revealed average interplanar distances of 0.43 nm and 0.26 nm, respectively (Fig. 1B–D). Figure 2 shows low-magnification TEM image of Ag-MNP (A), with corresponding TEM-EDS maps of iron (B), oxygen (C), and silver (D). In addition, spectral analysis (Fig. 2E) showed two optical observation peaks of Ag at 2.6 and 3 keV, which were distinctive absorption peaks of metallic silver nanocrystallities^28^. In Fig. 2F, the XRD pattern analysis of the Ag-MNP nanocomposite indicated the presence of silver (Ag) with four 2θ peaks at 37.9°, 44.2°, 64.3° and 77.2°, Fe_3_O_4_ (Magnetite) with six peaks at 30.1°, 35.4°, 43.1°, 56.9°, 62.5° and 74.9°, and FeO_2_ (Goethite) with three peaks at 21.1°, 33.1°, and 53.9°. Finally, the XRD analysis indicated that our Ag-MNP nanocomposite is composed of 58.6 Wt% (weight percent) of Fe_3_O_4_, 36.4 Wt% of FeO_2_, and 5 Wt% of Ag.

### Bacteria growth measurements

**Figure 3:**
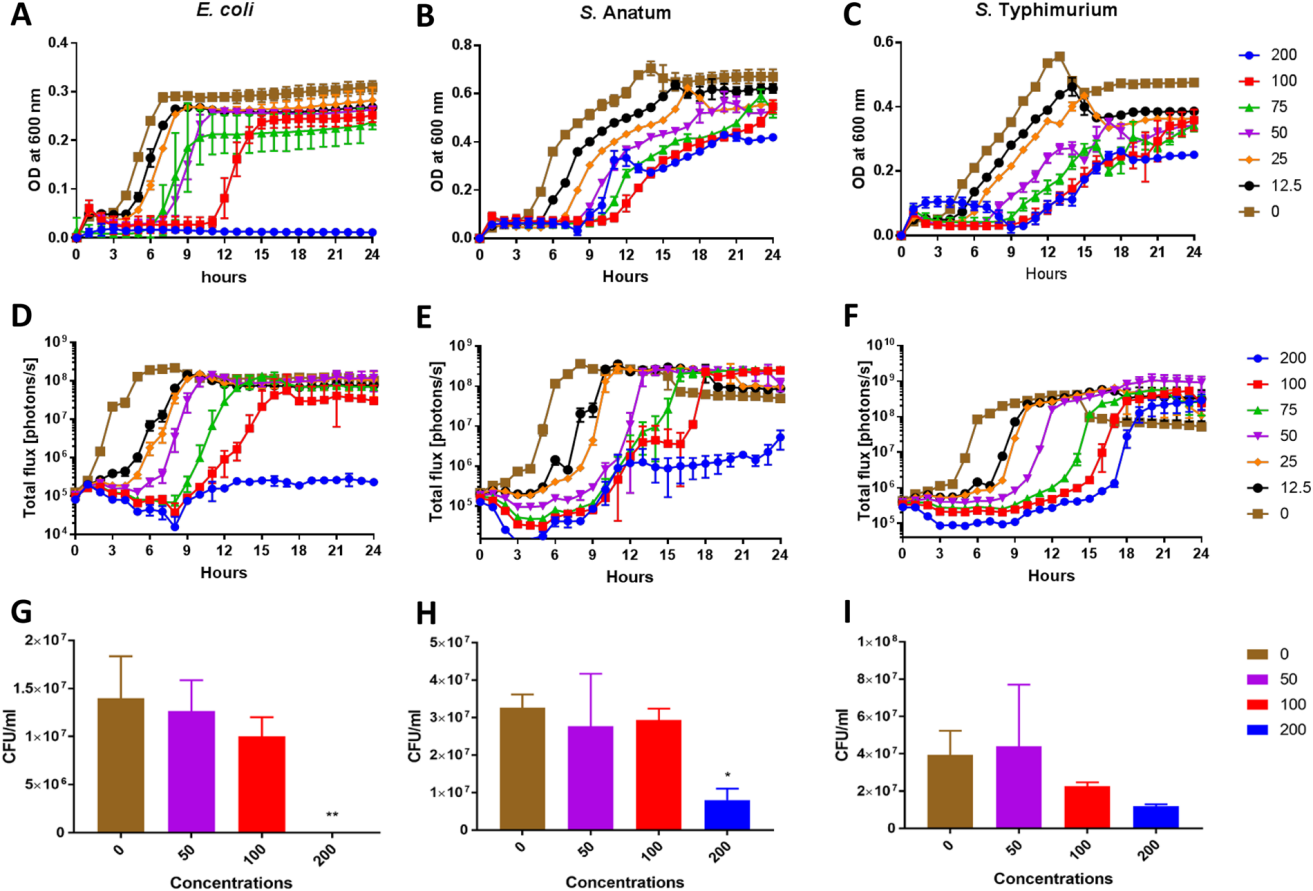
Growth curve profiles of *E. coli* (**A**,**D**,**G**), *S*. Anatum (**B**,**E**,**H**) and *S*. Typhimurium (**C**,**F**,**I**) exposed to various concentrations of silver-coated magnetic nanocomposites (Ag-MNP). Growth curves were generated by measurement of optical density at 600 nm (**A**–**C**) and bioluminescent imaging (**D**–**F**) for 24 h. The colony forming units (CFU)/ml were measured from each culture of *E. coli* (**G**), *S*. Anatum (**H**) and *S*. Typhimurium (**I**) after 24 h incubation with various Ag-MNP concentrations. Data are mean ± SD of three independent replicates (*P < 0.05 and **P < 0.01).

Figure 3 summarizes the bacterial growth performance in the presence of Ag-MNP (0, 12.5, 25, 50, 75, 100, and 200 µg/ml). Both optical density (Fig. 3A–C) and bioluminescent imaging (Fig. 3D–F) revealed dose-dependent growth inhibition of all bacteria strains (*E. coli* O157:H7, *S*. Typhimurium and *S*. Anatum). Regardless of the bacteria strain, the presence of Ag-MNP induced dose-dependent extended lag phases of bacterial growth, while the inhibitory patterns of bacteria growth curves varied with the Ag-MNP concentrations. Both optical density and bioluminescence emission measurements revealed the total inhibition of *E. coli* with 200 µg/ml. The Ag-MNP inducing dose-dependent inhibition was manifest with *E. coli*.

The antibacterial effect of Ag-MNP was confirmed with the CFU counts, following 24 hours post-culture on a solid phase (Fig. 3G–I). A dose-dependent decrease of CFU/ml was observed with *E. coli*, from 1.5 × 10^7^ CFU/ml in the control to a total absence in the 200 µg/ml Ag-MNP group (P < 0.01). A slightly similar inhibitory trend was observed with the other bacteria strains, and the 200 µg/ml Ag-MNP was confirmed as the most potent inhibitory concentration (P < 0.05).

### Morphology of *E. coli* O157:H7 under silver-coated magnetic nanocomposites

**Figure 4:**
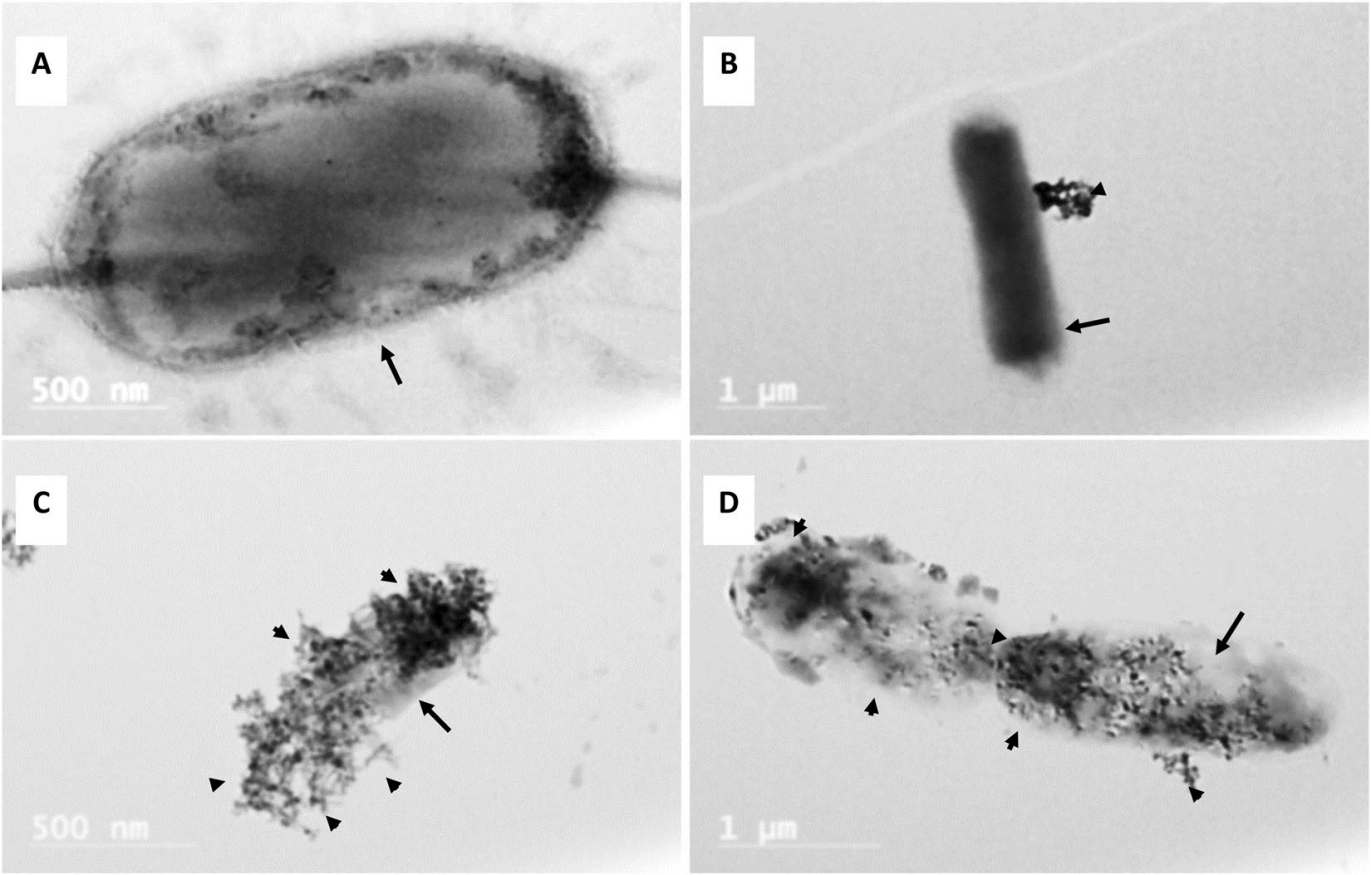
Transmission electron microscopy (TEM) images of *E. coli* interaction with Ag-MNP. Images show control *E. coli* with viable pili and flagella (**A**), a cluster of Ag-MNP attached to *E. coli* (**B**), *E. coli* surrounded by Ag-MNP (**C**), and irregular cell margin with infiltrated Ag-MNP (**D**). Arrows indicate bacterial membrane and arrowheads, the localization of Ag-MNP. Additional validation of *E*. *coli* and Ag-MNP interaction are shown in Supplementary Fig. 1.

The *E. coli* O157:H7 was selected for this study because of their higher sensitivity to Ag-MNP. *E. coli* were cultured in the presence of (0 or 100 µg/ml) Ag-MNP for TEM imaging. Control bacteria are shown in Fig. 4A, while the presence of Ag-MNP induced partial (Fig. 4B), complete surrounding (Fig. 4C), and total penetration of targeted bacteria leading to cell membrane destruction and cytoplasmic vacuolization (Fig. 4D; Arrow). The attachment of Ag-MNP was further confirmed by EDS analysis (Supplementary Fig. 1). The results exhibit Ag, Fe and O as the major components of Ag-MNP that are firmly attached to the surface of *E. coli*. The other *E. coli* cell showed the loss of membrane integrity and damaged shape by Ag-MNP (Fig. 4).

### Biocompatibility of the synthesized Ag-MNP

**Figure 5:**
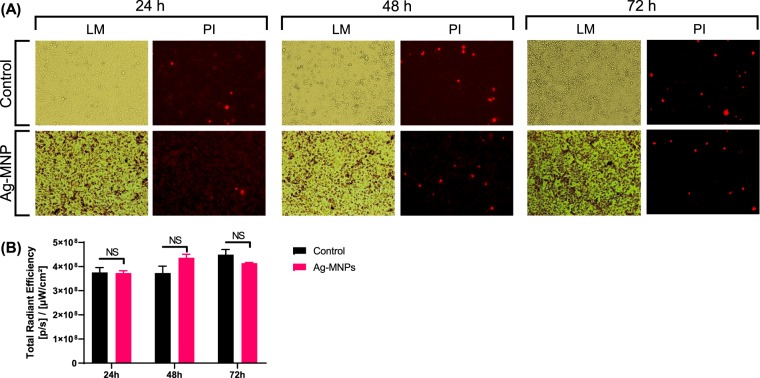
*In vitro* cytotoxicity assessment of Ag-MNP. Hep-2 cells were cultured for 24, 48 and 72 h in EMEM-Glutamine + 10% FBS medium containing 0 *μ*g/ml (Control) or 100 *μ*g/ml of Ag-MNP. Micrographs in (**A**) indicate Light Microscopy (LM) and fluorescence imaging of Propidium Iodide (PI; ʎ_Ex_ = 535 nm and ʎ_Em_ = 617 nm) that stained dead cells (Red color. The PI signal intensities were quantified in (**B**) using the *In Vivo* Imaging System (IVIS, Perking Elmer).

Human cervix carcinoma or HEP-2 cell line were culture in the presence of 0 (control) or 100 µg/ml (treatment) of Ag-MNP, which concentration showed stronger antibacterial effects in *E. coli*. Observation under light microscope indicated a consistent growth of cells in both control and treatment groups throughout culture (24, 48 and 72 h - Fig. 5A). The proportions of dead cells stained, with red-fluorescence PI, progressively increased during culture in both experimental groups (data not shown), and there were no significant differences between groups at each culture day (P > 0.05). Similar results were observed with in-cell fluorescence intensities of the PI within groups, which intensity is indicative of the extend of damages (Fig. 5B).

### Effects of Ag-MNP on protein changes

**Figure 6:**
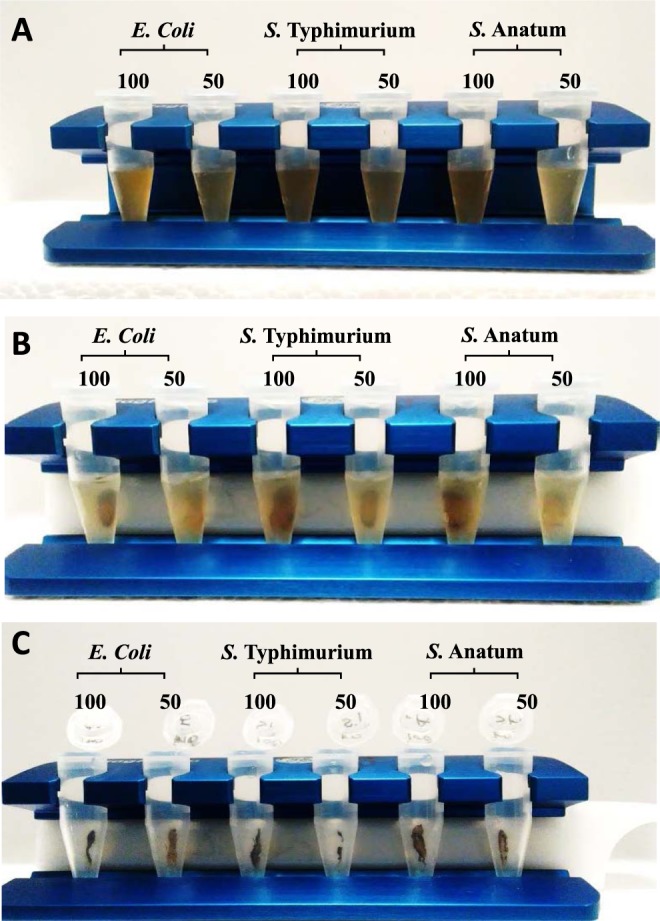
Separation of silver-coated magnetic nanocomposites (Ag-MNP). Bacteria were cultured in the presence of 0, 50, or 100 µg/ml of Ag-MNP (**A**). After 24 h incubation, bacteria were placed on a magnetic field to entrap free Ag-MNP and bacteria-Ag-MNP complexes (**B**), followed by the collection supernatant containing *E. coli* O157:H7, leaving entrapped Ag-MNP in tubes (**C**).

**Figure 7:**
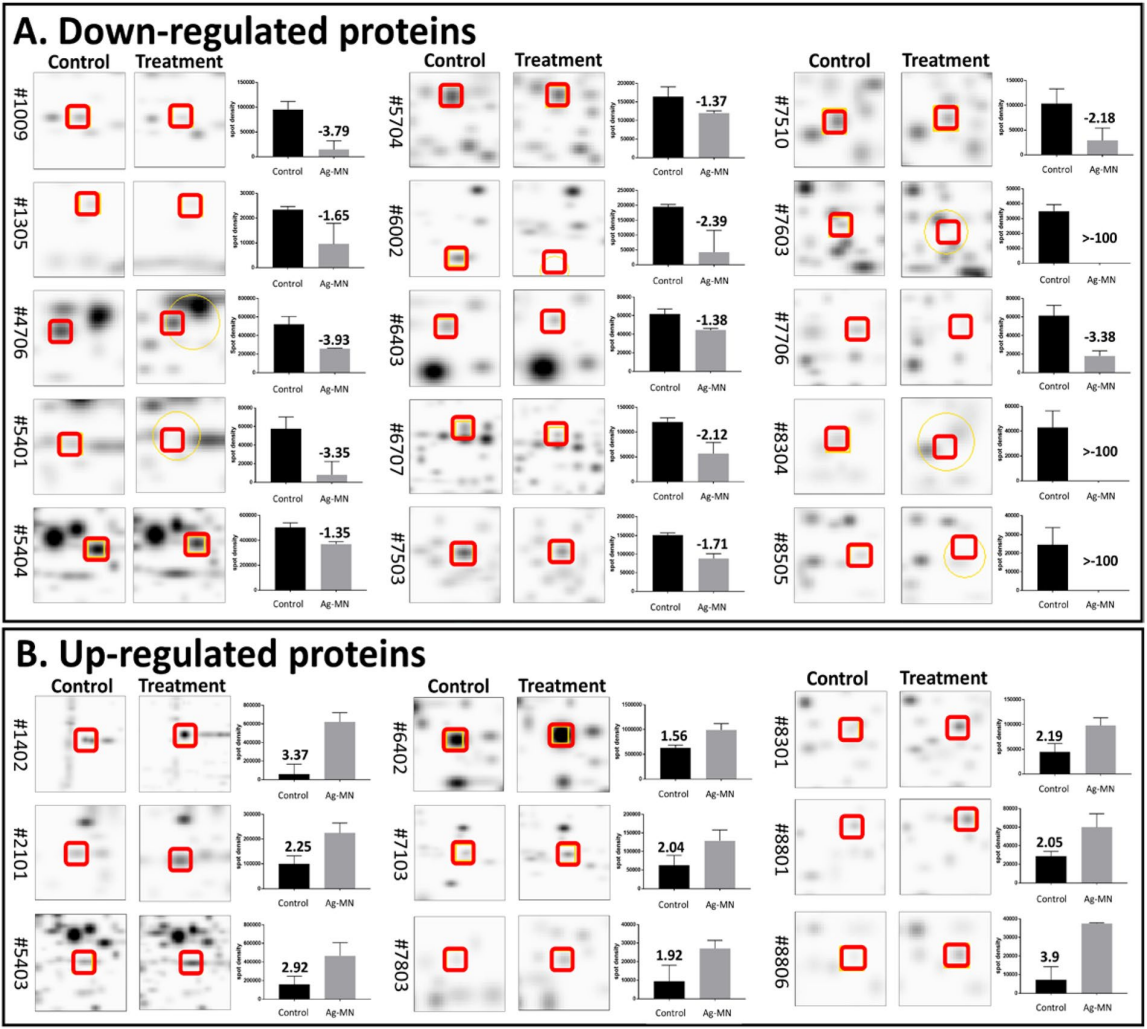
Quantitative comparisons of differentially expressed protein spots in control and Ag-MNP treated *E*. *coli* bacteria. The intensities of 15 down-regulated (**A**) and 9 up-regulated (**B**) spots were compared (PDQuest 2-D analysis) between the control and the treatment groups and plotted. Data are mean ± SD of three independent replicate gels (*p* < 0.05). Full-length representative gels are presented in Supplementary Fig. 2.

Following culture in 0 or 100 µg/ml of Ag-MNP and stoppage at the early stationary phase, *E. coli* O157:H7 were separated from Ag-MNP under a magnetic field (Fig. 6). Figure 7 shows representative gel electrophoreses of both control and Ag-MNP treated *E. coli* O157:H7, with a total of 372 spots that were detected across the gels (Supplementary Fig. 2). A total of 24 spots with significant intensity difference were detected (P < 0.05) and identified in Table 1. Fifteen were significantly down-regulated (Fig. 7A) versus nine up-regulated (Fig. 7B). The extreme effects were as over 100× down-regulation and 3× up-regulation.

**Figure 8:**
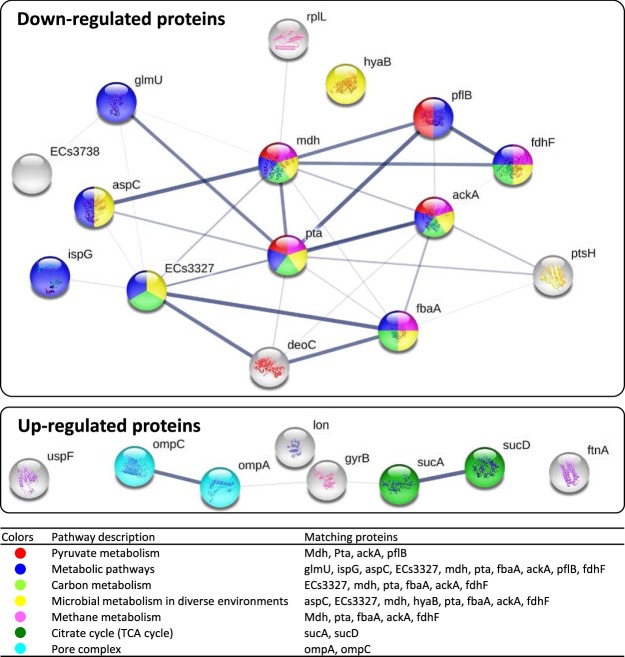
Interaction network of Ag-MNP regulated proteins in *E. coli*. Prediction was performed with STRING software (http://string-db.org) and networks were detected at *p* < 0.05. Seven networks were generated from 23 identified proteins that were significantly altered proteins in *E. coli* O157:H7 after treatment of Ag-MNP (pyruvate metabolism, metabolic pathways, carbon metabolism, microbial metabolism in diverse environments, methane metabolism, Citrate cycle, and pore complex).

All differentially detected proteins belonged to extracellular localization (pSORTb software) and were associated with: amino acid transport and metabolism (E, 40%), cell wall/membrane/envelope biogenesis (M, 21%), energy production and conversion (C, 21%), and lipid transport and metabolism (I, 9%) (COGs analysis). There were high protein interrelations within each up- and down-regulated groups (STRING software; Fig. 8). The presence of Ag-MNP altered the pyruvate metabolism, metabolic pathways, carbon metabolism, microbial metabolism in diverse environments, methane metabolism, TCA cycle, and pore complex pathways (STRING software; *P* < 0.05).

## Discussion

Silver nanoparticles have shown remarkable antibacterial effects against diverse microorganisms as part of efforts to develop the alternative agents for MDR bacteria^19^. Iron oxide nanoparticles have also displayed superior antibacterial activity against various pathogenic bacteria^13^, while the further combination of silver and iron oxide nanoparticles enhances their antibacterial and eco-friendly characteristics^27^. In the present study, we investigated the mechanism of antibacterial activity of Ag-MNP against foodborne bacteria especially in *E. coli* O157:H7.

The shape and size of nanoparticles are the key factors in antibacterial activities, and various shapes of silver nanoparticles contribute to achieve diversity in effective surface areas and active facet of silver nanoparticles^19^. The XRD analysis indicated that Ag-MNP is composed of Fe_3_O_4_ (Magnetite), FeO_2_ (Goethite), and Ag elemental. The attachment of Ag-MNP was confirmed by EDS and HR-TEM that exhibited the firm attachment of Ag-MNP to the surface of *E. coli* that led to cell damage and death. A further investigation of cytotoxicity of Ag-MNP was carried out by an analysis of the morphological changes in Ag-MNP treated Hep-2 cells, which indicated no significant differences (cell morphology and death) between treated and untreated cells after 24, 48 and 72 h. In a previous study, the incubation of eukaryote cells with nanoparticles of various shapes (spherical, rod, or cubic), within the range of 10–1,000 µg/ml, showed excellent biocompatibility without cytotoxicity effects^29^. Another study suggested that nanoparticles lesser than 50 nm in diameter have high stability, biocompatibility and antibacterial activity^30^. In the present study, Ag-MNP displayed peaks of typical absorption of silver, and two different types of nanocomposites, which may enhance the antibacterial effect of the synthesized Ag-MNP.

The antimicrobial activity of the synthesized Ag-MNP was tested on the growth kinetic of *E. coli*, *S*. Typhimurium, and *S*. Anatum, using optical density (OD), bioluminescence imaging (BLI), and colony forming unit (CFU) measurement. The dose-dependent delay of the lag phase and lower growth rate of all bacteria culminated with the strongest effect of 200 µg/ml, showing variability among bacteria. This result is indicative of an antibacterial activity of Ag-MNP that is dependent of the composition and structure of the bacterial membrane as previously suggested^31^. The TEM imaging revealed sequential effects of Ag-MNP on bacteria: membrane attachment, membrane penetration, and subsequent morphological changes. These effects are consistent with previous reports on silver nanoparticles binding to sulfur proteins of bacterial plasma membrane^32^, and causing severe disruption of membrane permeability, transmembrane transport systems, and intracellular homeostasis^31^. Additionally, bacteria exposure to iron oxide nanoparticles induce intracellular production of reactive oxygen species that damage a variety of cellular constituent, disrupt cell function, and eventually induce apoptosis^13^.

The inability to predict the affected molecules within the bacteria led us to perform a large-scale proteomic study to investigate the antibacterial mechanism of Ag-MNP. Here we used the *E. coli* strain because of its unpolluted growth kinetic response to Ag-MNP. The transfer of bacteria and Ag-MNP mixtures to a magnetic field showed high effectiveness in removing Ag-MNP nanoparticles from culture media. The proteomic findings strongly support previous reports, while indicating a wide range of proteins affected by the presence of Ag-MNP in the culture medium. Interestingly, these proteins have critical roles in various biological and physiological processes of the cell.

Numerous proteins were down regulated, and the *metabolic pathway* appeared as the mostly affected with numerous associated proteins found significantly inhibited. Among them, the phosphate acetyltransferase, malate dehydrogenase, formate acetyltransferase, acetate kinase, deoxyribose phosphate aldorase, hydrogenase-1, and formate dehydrogenase are known as key molecules for energy generation within the TCA cycle, glycolysis or gluconeogenesis^33–38^. Furthermore, the down-regulation of the phosphorcarrier protein HPr, an essential component of the sugar transporting phosphotransferase system, may suggest a deactivation of the *carbohydrate metabolism*^39^. The down-regulation of formate dehydrogenase and transketolase is crucial for the *control of cellular oxidative stresses*^40,41^. The aminotransferase protein is involved in the bacterial chromosome replication and cell division through its direct effect on the *regulation of bacterial cell cycle*^42^. The inhibition of this protein in our study may explain the dose-dependent extended lag phase of observed growth curves. Among the down-regulated proteins, the inhibition of the putative lipoprotein and biofunctional protein glmU may reduce *cell membrane integrity*. Indeed, the putative lipoprotein cross-links the bacterial outer membrane to the peptidoglycan lattice, which contributes to maintain the membrane stability^43^, while the glmU is involved in the synthesis of UDP-*N*-acetylglucosamine, an intermediate in the peptidoglycan and lipopolysaccharide biosynthetic pathway^44^. Furthermore, the bacteria exposure to Ag-MNP led to down-regulation of the 50 S ribosomal protein L7/L12, responsible for the translation and protein synthesis by catalyzing peptide bond formation^45^, and the 4-hydroxy-3-methybut-2-en-1-yldiphophate synthase (flavodoxin) protecting against DNA oxidative damage^46^. It is expected that these later effects of Ag-MNP contribute to the destabilization of bacterial membrane permeability, while denaturating DNA and deactivating protein synthesis.

In contrast, numerous proteins were up-regulated, and may constitute the adaptive responses elicited by the bacteria facing lethal stresses (i.e., nutrient starvation, oxidative stress, and membrane disruption^47^). The levels of bacterial non-heme protein C, universal stress protein F, lon protease, and DNA gyrase B were significantly elevated, more likely to cope with lethal stresses induced by Ag-MNP. The Ag-MNP seems to cause the up-regulation of bacterial non-heme protein C that is responsible for the iron detoxification and the removal of iron from the bacterial cytoplasm^48^. In addition, the universal stress protein F initiates the global gene regulations for biofilm formation, growth arrest and DNA protection^49^, and its up-regulation in this study could be crucial to manage environmental stressors such as metal ions and oxidative stress, which increase induces accumulation of inorganic polyphosphate (poly P) and forms a complex with lon protease to supply amino acids^50^. The elevation of DNA gyrase B, however, indicates a likely recovery of DNA molecules from cytotoxic oxidative stress^51^. Also, the upregulation of succinate CoA ligase subunit alpha (sucD) and 2-oxoglutarate dehydrogenase E1 component (sucA) playing crucial roles in the reduction of free radical formation and protection mitochondria from oxidative damage^52^ was also observed. In the other hand, the outer membrane protein A and C (ompA and ompC) were significantly increased, which was in accordance with a previous study reporting similar accumulations in *E. coli* exposed to silver nanoparticles^53^. These authors attributed this increase to the loss of proton motive force and intracellular ATP. Taken together, the proteomic findings suggest that Ag-MNP induce inhibition of metabolic pathways for energy production, which ultimately leads to the alteration of bacterial membrane structure and permeability, loss of cellular contents, and impairment of membrane transport. Further studies such as genomics and metabolomics will provide a valuable insight into the precise mechanism of antibacterial action of Ag-MNP.

To summarize, the present study describes the antibacterial effects of Ag-MNP using growth kinetics, TEM analysis and proteomic approach. Ag-MNP showed the significant growth inhibition in three foodborne bacteria, and TEM analysis of *E. coli* displayed the attachment and penetration of Ag-MNP finally leading to morphological changes such as vacuole formation and rupture of cells. The Ag-MNP induced global changes in the overall proteomes of *E. coli*, which demonstrated disruption of energy metabolism, inhibition of protein synthesis, interference with DNA synthesis and alteration of cell membrane integrity. Based on the evident antibacterial mechanism, this study suggests that Ag-MNP has the antibacterial capacity and could be an effective treatment against *E. coli* O157:H7.

## Methods

### Properties of silver-coated magnetic nanocomposites

Iron oxide or magnetic nanoparticles (MNP) were synthesized under a non-disclosed Intellectual Property by Clemente Associates (Madison, CT, USA). Briefly, coating with dextran further allowed for the attachment of silver ions to form a stable silver-coated magnetic nanocomposites (Ag-MNP). Stock solution (1 mg/ml = ~2.5 × 10^6^ particles/mg) containing 0.02% sodium azide was stored at 4 °C during experiments.

### Characterization of synthesized Ag-MNP and their interactions with bacteria

The synthesized Ag-MNP nanocomposites were resuspended in water and subsequently deposited onto a formvar/carbon-coated TEM grid, followed by high-resolution transmission electron microscopy imaging (HR-TEM 2100 at 200 kV: JEOL; Peabody, MA, USA) to assess the shape and size of the Ag-MNP. The binding of Ag ions to the synthesized iron oxide core was imaged with energy dispersive X-ray spectrometry (EDS: Oxford instruments; Concord, MA, USA). The X-ray diffraction (XRD) measurement was carried out by Rigaku X-ray diffractometer (Rigaku’s Ultima III XRD; The Woodlands, TX, USA) with Cu Kα X-ray source (*λ* = 1.54056 Å) at a generator voltage 40 kV, a generator current 44 mA with the scanning rate 1°/min.

Interactions between Ag-MNP and bacteria were performed after a co-culture of 24 hours. Bacteria were fixed with 2.5% glutaraldehyde, centrifuged, re-suspended in distilled water, stained with 1% uranyl acetate, and loaded onto a 100 mesh copper TEM grid. A total of five independent Ag-MNP preparations was performed. HR-TEM and EDS analysis were performed to determine the morphological changes in *E. coli* by Ag-MNP.

### Bacteria transformation and selection

Three foodborne pathogens, *E. coli* O157:H7 (ATCC 43888), *Salmonella enterica* serovar Anatum (ATCC 9270) and *Salmonella enterica* serovar Typhimurium (ATCC 14028) were cultured on Luria-Bertani (LB) agar (Becton Dickinson: Franklin Lakes; NJ, USA) or in LB broth with shaking at 37 °C. Bacteria were transformed with plasmid pXen 13-*luxCDABE* (Caliper Life Sciences; Hopkinton, MA, USA) by electroporation and resulting bioluminescent bacteria were selected in LB agar supplemented with Ampicillin (100 µg/ml); and successful transformation of growing bioluminescent bacteria was verified by *In Vivo* Imaging System (IVIS: Lumina XRMS Series III system; Perkin Elmer, Waltham, MA, USA).

### Bacteria growth analyses and colony forming unit counts

All bacteria strains were grown in LB broth supplemented with ampicillin until the early stationary phase and adjusted to 2 × 10^5^ CFU/ml in LB broth. Adjusted bacteria were incubated with various concentrations of Ag-MNP (0, 12.5, 25, 50, 75, 100 and 200 μl/ml) for 24 hours. Bacterial growth was measured every hour by OD_600_ (SpectraMax plus 384 spectrophotometer: Molecular Devices; San Jose, CA, USA) and bioluminescence imaging (IVIS).

Following the 24 hours culture mentioned above, aliquots of bacteria in each treatment group were sub-cultured for an additional 24 h culture in LB agar to determine the number of live bacteria (CFU/ml) in each culture plate.

### *In vitro* cytotoxicity assay

The cytotoxicity of Ag-MNP to Hep-2 (Human cervix carcinoma) cell was determined by staining with propidium iodide (PI) nucleic acid solution (Molecular Probes, Eugene, OR, USA). PI is a red-fluorescent nuclear and chromosome counterstaining dye. Since PI is excluded from healthy cell, it can be used to detect the cells with damaged plasma membrane or dead cells. The Hep2 cells (1 × 10^5^ cells/well) in 1 ml culture medium (Eagle’s minimum essential medium +2 mM Glutamine +10% fetal bovine serum) were seeded into a 12-well plate. After overnight incubation at 37 °C in 5% CO_2_ humidified atmosphere, the media from the 12 well plates were replaced the media containing 100 µg/ml of Ag-MNP, which showed the effective antimicrobial effects. The cells were further incubated for 24 and 48 h. At the end of each incubation period, the media were replaced with PBS containing 1 µg/ml of PI and the plates were incubated for 30 min. The morphological changes and PI signals were determined by EVOS FL auto cell imaging system (Thermo Fisher Scientific, Waltham, MA, USA). Total radiance efficiency from the wells was measured by IVIS system. Statistical analyses were carried out using Graphpad Prism version 8.1.1. (La Jolla, CA, USA). Significant differences were determined by Student’s *t* test. Data were presented as mean ± standard deviation (SD).

### Two-dimensional electrophoresis and protein spot excision

All bacteria strains were grown in LB broth containing 0, 50, and 100 µg/ml of Ag-MNP up to early stationary phase (~18 hours). Bacterial suspensions were transferred into Eppendorf tubes and placed under a magnetic field to allow the entrapping of Ag-MNP (free or attached). Thereafter, the remaining bacterial suspensions was eluted, washed three times by successive centrifugations (13,000 × *g*) after resuspensions with PBS. Pelleted bacteria were used for the proteomic study.

Total bacterial protein was extracted using Gene elute RNA/DNA/Protein purification plus kit (Sigma-Aldrich; St. Louis, MO, USA) and precipitated with TCA/Acetone. Resulting pellets were dissolved in rehydration sample buffer (7 M urea, 2 M thiourea, 4% CHAPS (w/v), and 20 mM dithiothreitol) and Isoelectric focusing (IEF) was carried out using 11-cm ReadyStrip IPG, pH4-7 (Bio-Rad; Hercules, CA, USA). The IEF conditions consisted of 30 V for 12 hours (rehydration), 500 V for 15 min, and 8,000 V for 2.5 hours with a total of 35 kVh. Thereafter, all strips were placed in the equilibration buffer (6 M urea, 20% glycerol, 2% SDS, and 0.375 M Tris-HCL pH6.8) containing 2% dithiothreitol (w/v), for 15 min and transferred to a second equilibration buffer containing 2.5% iodoacetamide (w/v), for an additional 15 min. Each strip was relocated onto 4–20% a gradient pre-casting Criterion TGX Precast Gel (Bio-Rad; Hercules, CA, USA) to perform the second-dimensional electrophoresis. Thereafter, the gels were stained with Bio-safe Coomassie G-250 solution (Bio-Rad; Hercules, CA, USA).

Three replicated gels of bacteria cultured with Ag-MNP (0 or 100 µg/ml) were generated and scanned with Proteome Works Plus Spot Cutter (Bio-Rad; Hercules, CA, USA). Resulting images were aligned by PDQuest 2-D analysis software, version 7 (Bio-Rad; Hercules, CA, USA) and differentially detected spots were excised for protein identification.

### In-gel protein digestion and identification

Twenty-four spots containing protein targets were excised and subjected to in-gel digestion using the In-gel tryptic digestion kit (Thermo Scientific; Rockford, IL, USA). Extracted proteins/peptides were analyzed by Orbitrap LTQ Velos mass spectrometer (Thermo Fisher Scientific; Waltham, MA, USA) combined with the UltiMate 3000 nanoflow HPLC system (Thermo Fisher Scientific) to collect spectral data. Briefly, samples were loaded on a reversed-phase fused silica Acclaim PepMap C18 column (75 µm × 150 mm, Thermo Fisher Scientific; Rockford, IL, USA); peptides were separated by a constant flow rate (0.3 µl/min) in a 60 min long linear gradient of acetonitrile in 0.1% formic acid, 2–55% for 35 min, 95% for 10 min, and 2% for 15 min; and peptides were subsequently collected by linear trap mass spectrometer. The mass spectra were obtained in a data dependent acquisition mode with dynamic exclusion being applied in 8 scan events: one MS scan (300–2,000 m/z range) followed by seven tandem mass spectrometry (MS/MS) scans for the most intense ion detected in the scan. Other parameters were set as follows: Normalized collision energy: 35%; automatic gain control “on: with MSn target 4 × 104, isolation width (m/z): 1.5, capillary temperature 170 °C, and spray voltage 1.97 kV.

The raw files were examined by the SEQUEST HT algorithm of the Proteome Discoverer 2.1 SP1 software (Thermo Fisher Scientific; Rockford, IL, USA) using the following parameters: lowest and highest charge: +1 and +3; minimum and maximum precursor mass: 300 and 6,000 Da; minimum S/N ratio: 3; enzyme: trypsin; maximum missed cleavages: 2; dynamic modification: cysteine carbamidomethylation (+57.021), methionine oxidation (+15.995), and methionine dioxidation (+31.990). Spectral data were matched with *E. coli* O157:H7 protein database from the National Center for Biotechnology Information (www.ncbi.nlm.nih.gov) to generate the Proteome Discoverer results files (.msf) that served as a decoy database.

### Bioinformatics analyses

Subcellular localization of identified protein was predicted using the pSORTb software version 3 (http://www.psort.org/psortb/). The functional annotation and classification of biological process was carried out by the COGs (Clusters of Orthologous Groups) analysis (https://www.ncbi.nlm.nih.gov/COG/). Protein interaction network and pathways were analyzed by the STRING software version 10.5 (https://string-db.org/).

### Statistical analyses

All experiments were repeated at least three times. A two-tailed nonparametric and Turkey’s multiple comparison tests were used to analyze bacteria growth and colony forming unit data (GraphPad Prism V7.0.1; La Jolla, CA, USA). All detected spots on gel electrophoreses were normalized, and significantly differentially expressed spots (increased or decreased) were compared by Student’s *t*-test (PDQuest 2-D analysis software, V7). Following MS/MS, proteins exhibiting at least three spectral counts were considered, and their detections were validated (false discovery rate less than 1%) with ProteoIQ 2.1 software (Premier Biosoft; Palo Alto, CA, USA). All data are expressed as mean ± standard deviation (SD) with P < 0.05 set as the threshold of significant differences.

## Supplementary information

High Resolution (HR) TEM and Energy Dispersive Spectrometry (EDS) analyses of E. coli incubated with Ag-MNP (100 µg/ml).

Representative two-dimensional electrophoresis of Control (A) and Ag-MNP treated (B) E. coli.

## References

[1] Desselberger U (2000). Emerging and re-emerging infectious diseases. J. Infect..

[2] Kumar, S., Mukherjee, M. M. & Varela, M. F. Modulation of bacterial multidrug resistance efflux pumps of the major facilitator superfamily. *Int. J. Bacteriol*. **2013** (2013). 10.1155/2013/204141 PMC4347946 25750934

[3] Paterson GK, Harrison EM, Holmes MA (2014). The emergence of mecC methicillin-resistant *Staphylococcus aureus*. Trends Microbiol..

[4] O’Driscoll T, Crank CW (2015). Vancomycin-resistant enterococcal infections: epidemiology, clinical manifestations, and optimal management. Infect. Drug resist..

[5] Gu H, Ho P, Tong E, Wang L, Xu B (2003). Presenting vancomycin on nanoparticles to enhance antimicrobial activities. Nano letters.

[6] Zhao Y (2010). Small molecule-capped gold nanoparticles as potent antibacterial agents that target gram-negative bacteria. J. Am. Chem. Soc..

[7] Sondi I, Salopek-Sondi B (2004). Silver nanoparticles as antimicrobial agent: a case study on *E. coli* as a model for Gram-negative bacteria. J. Colloid Interface Sci..

[8] Kang S, Herzberg M, Rodrigues DF, Elimelech M (2008). Antibacterial effects of carbon nanotubes: size does matter!. Langmuir.

[9] Makarovsky I (2011). Novel triclosan‐bound hybrid‐silica nanoparticles and their enhanced antimicrobial properties. Adv. Funct. Mater..

[10] Espitia PJP (2012). Zinc oxide nanoparticles: synthesis, antimicrobial activity and food packaging applications. Food Bioprocess Tech..

[11] Martinez-Gutierrez F (2010). Synthesis, characterization, and evaluation of antimicrobial and cytotoxic effect of silver and titanium nanoparticles. Nanomed_Nanotechnol..

[12] Kruk T, Szczepanowicz K, Stefańska J, Socha RP, Warszyński P (2015). Synthesis and antimicrobial activity of monodisperse copper nanoparticles. Colloids Surf. B.

[13] Li Y (2018). The detailed bactericidal process of ferric oxide nanoparticles on *E. coli*. Molecules.

[14] Khan A, Rashid R, Murtaza G, Zahra A (2014). Gold nanoparticles: synthesis and applications in drug delivery. Trop. J. Pharm. Res..

[15] Dinali R, Ebrahiminezhad A, Manley-Harris M, Ghasemi Y, Berenjian A (2017). Iron oxide nanoparticles in modern microbiology and biotechnology. Crit. Rev. Microbiol..

[16] Nagasundaram N, Rahuman M, Raghavan P (2014). Antibacterial application studies of nanosilver incorporated products. Int. J. Pharm. Res. Bio–Sci..

[17] Chaloupka K, Malam Y, Seifalian AM (2010). Nanosilver as a new generation of nanoproduct in biomedical applications. Trends Biotechnol..

[18] Marambio-Jones C, Hoek EM (2010). A review of the antibacterial effects of silver nanomaterials and potential implications for human health and the environment. J. Nanopart. Res..

[19] Dakal TC, Kumar A, Majumdar RS, Yadav V (2016). Mechanistic basis of antimicrobial actions of silver nanoparticles. Front. Microbiol..

[20] Feng Q (2000). A mechanistic study of the antibacterial effect of silver ions on *Escherichia coli* and *Staphylococcus aureus*. J. Biomed. Mater. Res..

[21] Holt KB, Bard AJ (2005). Interaction of silver (I) ions with the respiratory chain of *Escherichia coli*: an electrochemical and scanning electrochemical microscopy study of the antimicrobial mechanism of micromolar Ag+. Biochemistry.

[22] Yavuz CT (2006). Low-field magnetic separation of monodisperse Fe_3_O_4_ nanocrystals. Science.

[23] Bock N (2010). A novel route in bone tissue engineering: magnetic biomimetic scaffolds. Acta Biomaterialia.

[24] Mahmoudi M, Simchi A, Imani M (2010). Recent advances in surface engineering of superparamagnetic iron oxide nanoparticles for biomedical applications. J. Iran. Chem. Soc..

[25] Mahmoudi M, Sant S, Wang B, Laurent S, Sen T (2011). Superparamagnetic iron oxide nanoparticles (SPIONs): development, surface modification and applications in chemotherapy. Adv. Drug Deliv. Rev..

[26] Mahmoudi M (2010). Magnetic resonance imaging tracking of stem cells *in vivo* using iron oxide nanoparticles as a tool for the advancement of clinical regenerative medicine. Chem. Rev..

[27] Yong C, Chen X, Xiang Q, Li Q, Xing X (2018). Recyclable magnetite-silver heterodimer nanocomposites with durable antibacterial performance. Bioactive materials.

[28] Magudapathy P, Gangopadhyay P, Panigrahi B, Nair K, Dhara S (2001). Electrical transport studies of Ag nanoclusters embedded in glass matrix. Physica B: Condensed Matter.

[29] Zhou X (2012). Controllable synthesis, magnetic and biocompatible properties of Fe_3_O_4_ and α-Fe_2_O_3_ nanocrystals. J. Solid State Chem..

[30] Yacamán MJ, Ascencio J, Liu H, Gardea-Torresdey J (2001). Structure shape and stability of nanometric sized particles. J. Vac. Sci. Technol. B Microelectron. Nanometer Struct. Process. Meas. Phenom..

[31] Kim JS (2007). Antimicrobial effects of silver nanoparticles. Nanomed_ Nanotechnol..

[32] Pal S, Tak YK, Song JM (2007). Does the antibacterial activity of silver nanoparticles depend on the shape of the nanoparticle? A study of the gram-negative bacterium Escherichia coli. Appl. Environ. Microbiol..

[33] Bae YM, Yoon JH, Kim JY, Lee SY (2018). Identifying the mechanism of *Escherichia coli* O157: H7 survival by the addition of salt in the treatment with organic acids. J. Appl. Microbiol..

[34] Heine A, Luz JG, Wong C-H, Wilson IA (2004). Analysis of the class I aldolase binding site architecture based on the crystal structure of 2-deoxyribose-5-phosphate aldolase at 0.99 Å resolution. J. Mol. Biol..

[35] Kakuda H, Hosono K, Ichihara S (1994). Identification and characterization of the ackA (acetate kinase A)-pta (phosphotransacetylase) operon and complementation analysis of acetate utilization by an ackA-pta deletion mutant of *Escherichia coli*. J. Biochem..

[36] Macomber L, Elsey SP, Hausinger RP (2011). Fructose‐1, 6‐bisphosphate aldolase (class II) is the primary site of nickel toxicity in *Escherichia coli*. Mol. Microbiol..

[37] Makumire S, Revaprasadu N, Shonhai A (2015). DnaK protein alleviates toxicity induced by citrate-coated gold nanoparticles in *Escherichia coli*. PLoS One.

[38] Lukey MJ (2010). How *Escherichia coli* is equipped to oxidize hydrogen under different redox conditions. J. Biol. Chem..

[39] Rodionova IA (2017). The phosphocarrier protein HPr of the bacterial phosphotransferase system globally regulates energy metabolism by directly interacting with multiple enzymes in *Escherichia coli*. J. Biol. Chem., jbc..

[40] Domain F, Bina XR, Levy SB (2007). Transketolase A, an enzyme in central metabolism, derepresses the marRAB multiple antibiotic resistance operon of *Escherichia coli* by interaction with MarR. Mol. Microbiol..

[41] Iwadate Y, Funabasama N, Kato J-L (2017). Involvement of formate dehydrogenases in stationary phase oxidative stress tolerance in *Escherichia coli*. FEMS Microbiol. Lett..

[42] Liu F, Hao J, Yan H, Bach T, Fan L (2014). AspC-mediated aspartate metabolism coordinates the *Escherichia coli* cell cycle. PLoS One.

[43] Planchon M, Léger T, Spalla O, Huber G, Ferrari R (2017). Metabolomic and proteomic investigations of impacts of titanium dioxide nanoparticles on *Escherichia coli*. PLoS One.

[44] Stokes SS (2012). Inhibitors of the acetyltransferase domain of N-acetylglucosamine-1-phosphate-uridylyltransferase/glucosamine-1-phosphate-acetyltransferase (GlmU). Part 2: optimization of physical properties leading to antibacterial aryl sulfonamides. Bioorg. Med. Chem. Lett..

[45] Contreras A, VAZQUEZ D (1977). Cooperative and antagonistic interactions of peptidyl‐tRNA and antibiotics with bacterial ribosomes. Eur. J. Biochem..

[46] Moyano AJ (2014). A long-chain flavodoxin protects *Pseudomonas aeruginosa* from oxidative stress and host bacterial clearance. PLoS genetics.

[47] Poole K (2012). Bacterial stress responses as determinants of antimicrobial resistance. J. Antimicrob. Chemother..

[48] Smith JL (2004). The physiological role of ferritin-like compounds in bacteria. Crit. Rev. Microbiol..

[49] Kvint K, Nachin L, Diez A, Nyström T (2003). The bacterial universal stress protein: function and regulation. Curr. Opin. Microbiol..

[50] Kuroda A (2001). Role of inorganic polyphosphate in promoting ribosomal protein degradation by the Lon protease in *E. coli*. Science.

[51] Dwyer DJ, Kohanski MA, Hayete B, Collins JJ (2007). Gyrase inhibitors induce an oxidative damage cellular death pathway in *Escherichia coli*. Mol. Syst. Biol..

[52] McLain AL, Szweda PA, Szweda LI (2011). α-Ketoglutarate dehydrogenase: a mitochondrial redox sensor. Free Radic. Res..

[53] Lok CN (2006). Proteomic analysis of the mode of antibacterial action of silver nanoparticles. J. Proteome Res..

