# Supplementary material for: Silver-coated magnetic nanocomposites induce growth inhibition and protein changes in foodborne bacteria
Source: Sci Rep. 2019 Nov 25;9:17499. doi: 10.1038/s41598-019-53080-x (PMC6877574; doi:10.1038/s41598-019-53080-x)
Supplement: Supplementary file 1 — High Resolution (HR) TEM and Energy Dispersive Spectrometry (EDS) analyses of E. coli incubated with Ag-MNP (100 µg/ml). [file 41598_2019_53080_MOESM1_ESM.pdf]

Silver-coated magnetic nanocomposites induce growth inhibition and protein changes in foodborne bacteria

Seong B. Park[1], Shecoya B. White[2], Christy S. Steadman[1], Tibor Pechan[3], Olga Pechanova[3], Henry J. Clemente[4], Rooban VKG Thirumalai[5], Scott T. Willard[1,6], Peter L. Ryan[1,7], Jean M. Feugang[1,*]

Supplementary Figure 1. High Resolution (HR) TEM and Energy Dispersive Spectrometry (EDS) analyses of E. coli incubated with Ag-MNP (100 μg/ml). Micrograph A corresponds to HR-TEM, while EDS images of Iron, Oxygen, Silver, and Carbon distributions are shown in micrographs B, C, D, and E, respectively. Micrograph F shows the EDS spectrum of each Fe, Ag and Oxygen components of the Ag-MNP nanocomposite.

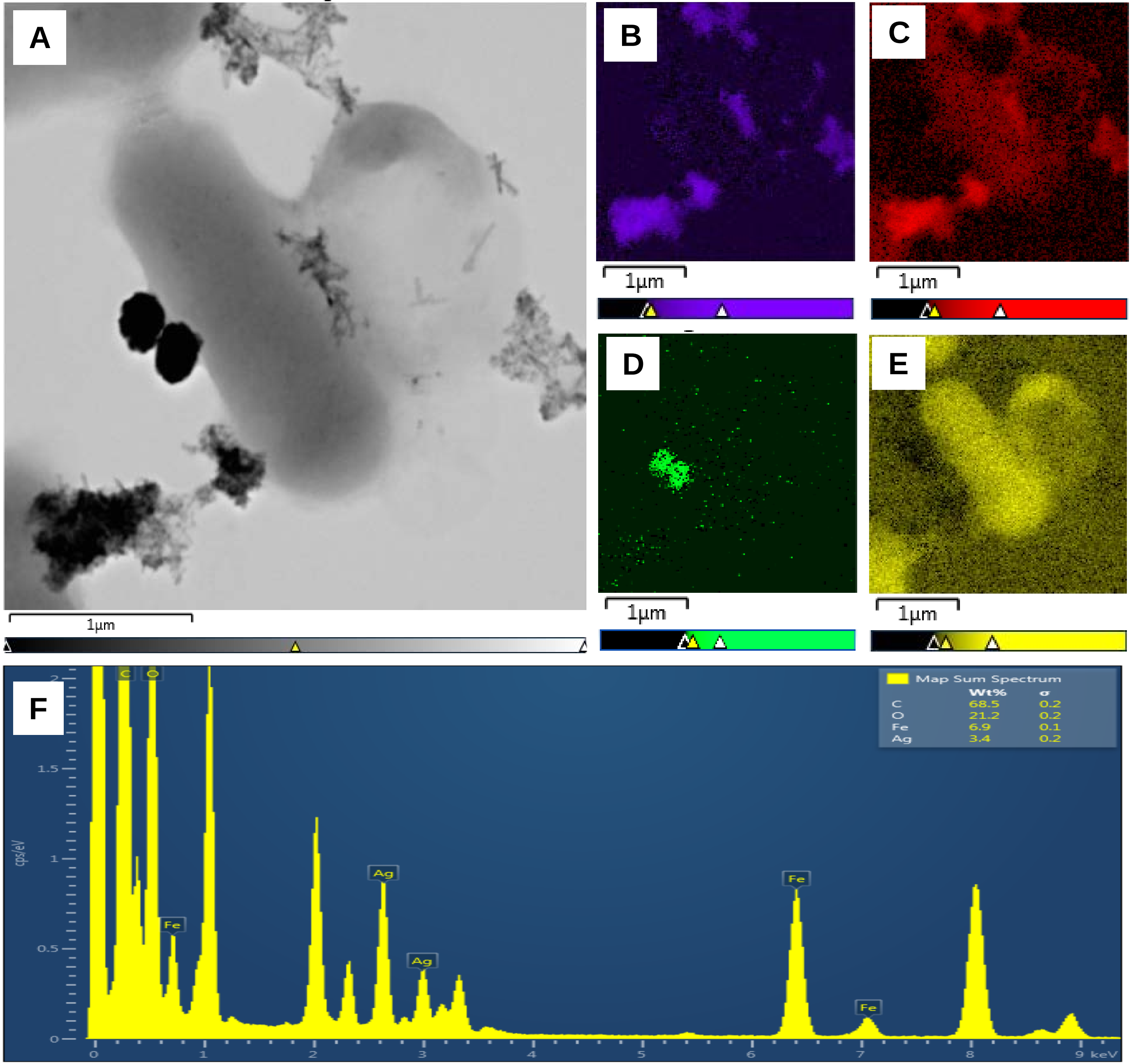

A
1μm
B
1μm
C
1μm
D
1μm
E
1μm
F
Map Sum Spectrum
Wt% σ
C 68.5 0.2
O 21.2 0.2
Fe 6.9 0.1
Ag 3.4 0.2
cps/eV
keV
