# Supplementary material for: Silver-coated magnetic nanocomposites induce growth inhibition and protein changes in foodborne bacteria
Source: Sci Rep. 2019 Nov 25;9:17499. doi: 10.1038/s41598-019-53080-x (PMC6877574; doi:10.1038/s41598-019-53080-x)
Supplement: Supplementary file 2 — Representative two-dimensional electrophoresis of Control (A) and Ag-MNP treated (B) E. coli. [file 41598_2019_53080_MOESM2_ESM.pdf]

Silver-coated magnetic nanocomposites induce growth inhibition and protein changes in foodborne bacteria

Seong B. Park[1], Shecoya B. White[2], Christy S. Steadman[1], Tibor Pechan[3], Olga Pechanova[3], Henry J. Clemente[4], Rooban VKG Thirumalai[5], Scott T. Willard[1,6], Peter L. Ryan[1,7], Jean M. Feugang[1,*]

Supplementary Figure 2. Representative two-dimensional electrophoresis of Control (A) and Ag-MNP treated (B) *E. coli*. Extracted protein samples were successively separated by isoelectric focusing (p*I* 4-7) and 4-20% gradient SDS-PAGE. Significantly ($p<0.05$) differentially detected protein spots are indicated with black (down-regulated) and purple (up-regulated) colored arrows in Micrograph A, while opposite indications are shown in Micrograph B (black = up-regulated and Purple = down-regulated).

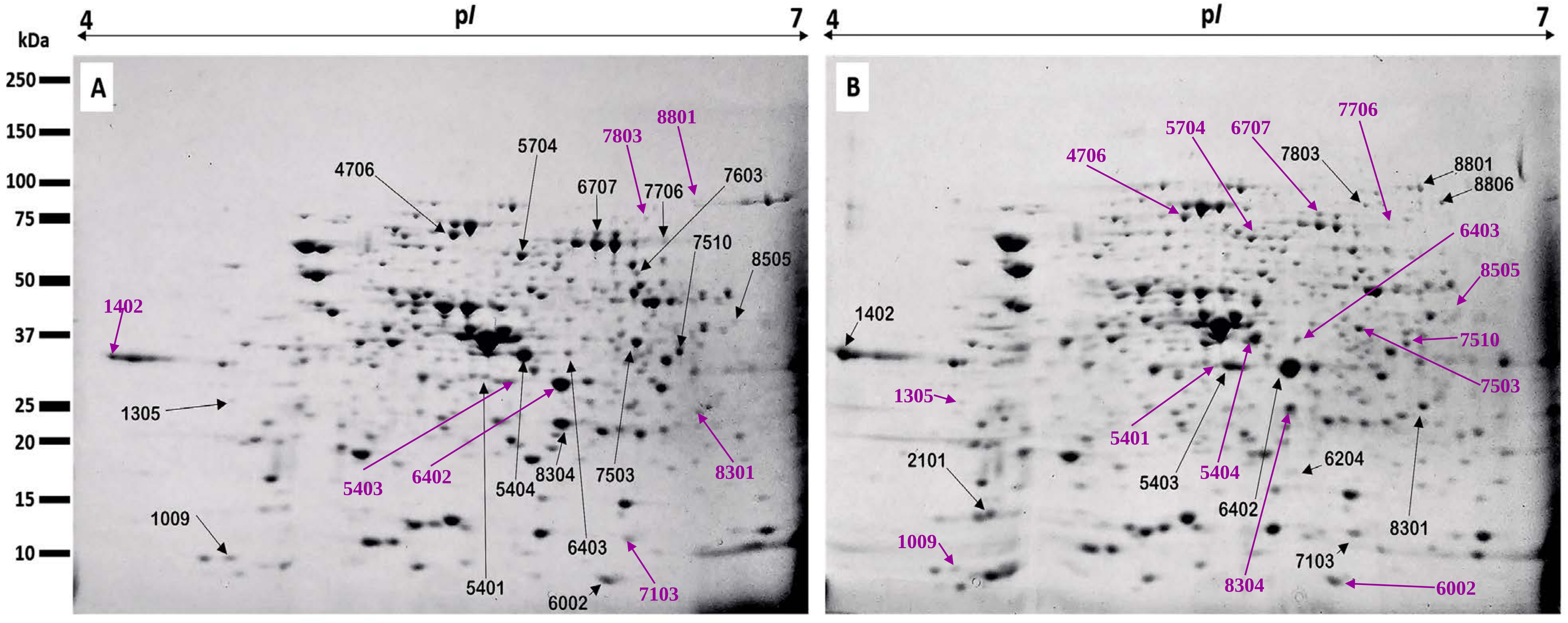

kDa
250
150
100
75
50
37
25
20
15
10
pI
4
7
A
B
1402
1305
1009
2101
4706
5704
6707
7803
7706
8801
8806
7603
7510
8505
6403
5401
5403
5404
6402
8304
8301
7503
7103
6002
6204
